# The Urology Residency Program in Israel—Results of a Residents Survey and Insights for the Future

**DOI:** 10.5041/RMMJ.10317

**Published:** 2017-10-16

**Authors:** Arnon Lavi, Sharon Tzemah, Anan Hussein, Ibrahim Bishara, Nikolay Shcherbakov, Genady Zelichenko, Alon Mashiah, Michael Gross, Michael Cohen

**Affiliations:** Department of Urology, Haamek Medical Center, Afula, Israel

**Keywords:** Residents, survey, training, urology residency

## Abstract

**Objective:**

Urology practice has undergone several changes in recent years mainly related to novel technologies introduced. We aimed to get the residents’ perspective on the current residency program in Israel and propose changes in it.

**Methods:**

A web-based survey was distributed among urology residents.

**Results:**

61 residents completed the survey out of 95 to whom it was sent (64% compliance). A total of 30% replied that the 9 months of mandatory general surgery rotation contributed to their training, 48% replied it should be shortened/canceled, and 43% replied that the Step A exam (a mandatory written certifying exam) in general surgery was relevant to their training. A total of 37% thought that surgical exposure during the residency was adequate, and 28% considered their training “hands-on.” Most non-junior residents (post-graduate year 3 and beyond) reported being able to perform simple procedures such as circumcision and transurethral resections but not complex procedures such as radical and laparoscopic procedures. A total of 41% of non-junior residents practice at a urology clinic. A total of 62% of residents from centers with no robotics replied its absence harmed their training, and 85% replied they would benefit from a robotics rotation. A total of 61% of residents from centers with robotics replied its presence harmed their training, and 72% replied they would benefit from an open surgery rotation. A total of 82% of the residents participated in post-graduate courses, and 81% replied they would engage in a clinical fellowship.

**Conclusion:**

Given the survey results we propose some changes to be considered in the residency program. These include changes in the general surgery rotation and exam, better surgical training, possible exchange rotations to expose residents to robotic and open surgery (depending on the availability of robotics in their center), greater out-patient urology clinic exposure, and possible changes in the basic science period.

## INTRODUCTION

The practice of urology has undergone enormous changes in recent years. These are mostly (but not only) related to rapid uptake of novel surgical technologies. Of these, a prominent change is the wide adoption of minimally invasive surgery in general and ongoing advancements in the field of endo-urology leading to miniaturization of surgical devices, hence stretching surgical bounds. The other side of the coin is the reduction in the volume of traditional open surgery. It is obvious that appropriate surgical training during urologic residency should follow and match these trends accordingly. This is not evident in the current urology residency program in Israel.

Another major change has been the working hours and labor limitations set by different regulatory bodies. These issued limits to weekly working hours and on-call shift durations, or imposed obligatory days-off after on-calls, leading to a significant cutback in the actual surgical and clinical exposure in the operating rooms, wards, and clinics.

The current urology program in Israel lasts 6 years and includes an obligatory 9-month general surgery rotation period, an elective 3-month rotation in related disciplines, and a 6-month period of basic science. Currently, residents take two written exams, referred to in Israel as Step A exams (*Shlav Alef*): the general surgery Step A exam and a urology Step A exam. This is followed by the Step B oral exam, which is taken only in urology.

In the USA, residency programs are constantly being evaluated. In 2013 the Accreditation Council for Graduate Medical Education (ACGME) issued the Next Accreditation System (NAS) to prepare physicians to practice in the twenty-first century.^1^ Urology was among the seven core specialties selected to implement it at the outset.^1^ The NAS set specific educational milestones to be met by residents in set intervals as they progress through their training. Moreover, the NAS requires annual data collection with corresponding review committee evaluations and 10-year accreditation visits. To date, programs in Israel have accreditation visits every 5 years, but no set milestones or timed program evaluations are conducted.

We present here the results of a survey performed among urology residents in Israel regarding issues related to the current residency program. Similar surveys on residents’ perspectives have been reported in other fields.^2^,^3^ We then set to review and point out suggested changes to be considered in the residency program according to the residents’ perspectives on the current program as reflected in the survey.

## METHODS AND RESULTS

We built a web-based survey comprising questions regarding different aspects of the current urology residency program in Israel. The translated survey used is brought in the Supplement. The survey was sent by e-mail to all registered residents in urology in Israel. At the time the survey was sent, 100 urology residents were registered. Out of the 95 to whom it was sent, 61 (64%) residents completed the survey (contact with 5 residents was not possible).

The first issue dealt with was the relevance of the current obligatory 9-month general surgery rotation. A total of 30% of responders replied that the general surgery rotation made a substantial contribution to their urology training. In contrast, 48% replied that the rotation should be shortened or abolished. Regarding the Step A exam in general surgery, 43% replied that the exam was relevant to their urology training.

**Figure 1:**
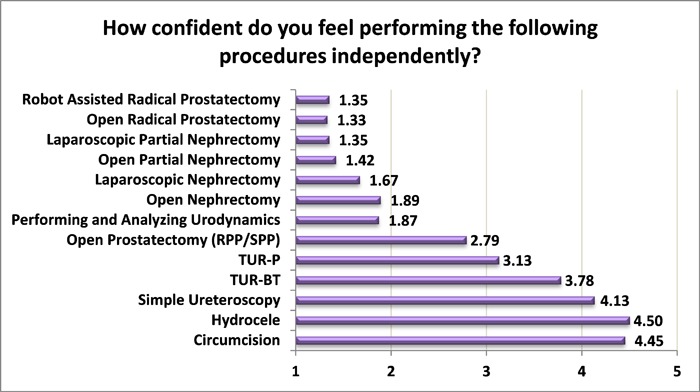
Degree of Confidence in Independently Performing Common Urologic Procedures Among Residents in Post-graduate Year 3 (PGY3) Confidence measured on a scale of 1 to 5 (1, not confident; 5, very confident). RPP, retropubic prostatectomy; SPP, suprapubic prostatectomy; TUR-BT, transurethral resection of bladder tumor; TUR-P, transurethral resection of prostate.

**Figure 2:**
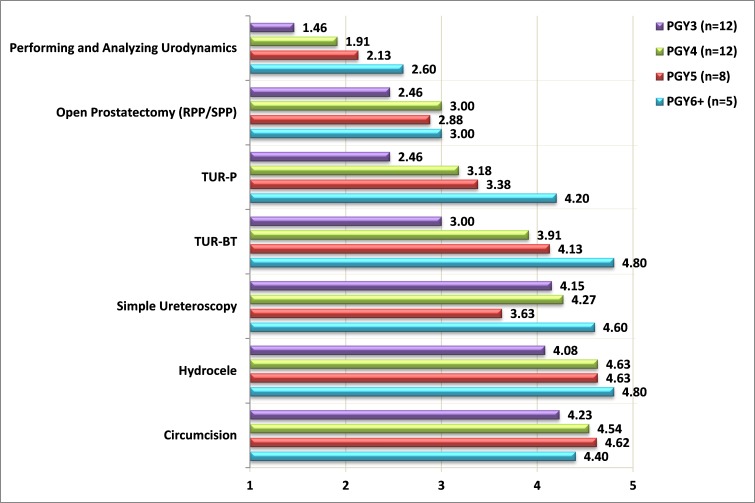
Degree of Confidence in Independently Performing Simple Urologic Procedures Among Residents in Post-graduate Year 3 (PGY3) and beyond Confidence measured on a scale of 1 to 5 (1, not confident; 5 very confident). PGY, post-graduate year; RPP, retropubic prostatectomy; SPP, suprapubic prostatectomy; TUR-BT, transurethral resection of bladder tumor; TUR-P, transurethral resection of prostate.

**Figure 3:**
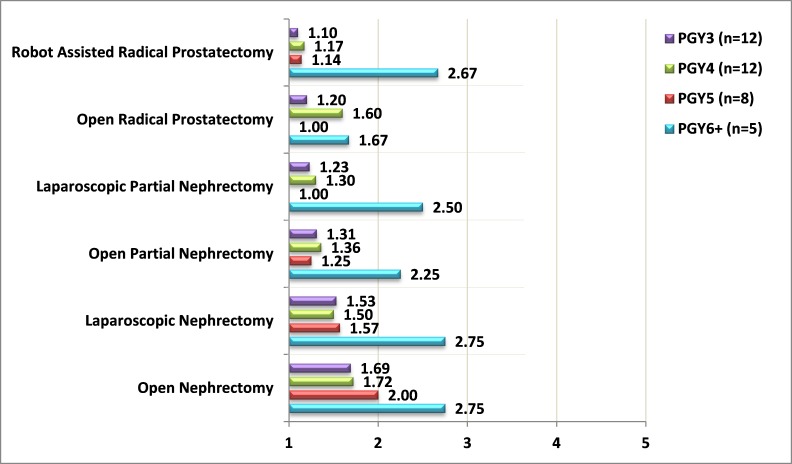
Degree of Confidence in Independently Performing Complex Urologic Procedures among Residents in Post-graduate Year 3 (PGY3) and beyond Confidence measured from 1 to 5 (1, not confident; 5, very confident).

Only 37% of the responders regarded the urologic surgical exposure during their residency as very good; 23% of post-graduate year (PGY) 1 residents considered the exposure to be very good, 30% among PGY2, 38% among PGY3, 50% among PGY4, 12.5% of PGY5, and 60% of PGY6. When the residents were asked if they considered their residency program as “hands-on,” only 28% replied positively. We then set to clarify what procedures the residents are confident enough to perform independently. We included standard procedures, most of which are considered urologic “bread and butter.” Only residents in PGY3 and beyond were presented with these questions. They were asked to rate their confidence in performing each procedure independently on a scale of 1 to 5, with 1 representing “not confident” and 5 representing “very confident.” Results are presented in Figures 1–3. It is evident that residents in general are not confident in performing complex procedures such as radical prostatectomies, nephrectomies, and laparoscopic and robotic procedures. It should be noted that except for a steep rise in PGY6, the degree of confidence in complex procedures does not rise as PGY advances. Nevertheless, even among PGY6 the degree of confidence in these complex procedures did not exceed 2.75 (on a scale of 1 to 5). In contrast, confidence does rise as PGY advances in simple procedures (hydrocele, circumcision, ureteroscopy, transurethral resections, and urodynamics). Only 5% of the residents replied being confident in performing a simple and routine procedure such as urodynamics.

Another important aspect during training is practicing in a urology clinic setting. Only 41% of non-junior residents (PGY3 and beyond) practice in a urology clinic routinely. Divided into PGY, 15% of PGY3 residents practice a clinic routinely, 17% of PGY4, 75% of PGY5, and 80% of PGY6.

To date, robotic surgery is available only in 7 of the 17 public medical centers in Israel with an approved urology residency program. Among residents from centers with no available robotic surgery, 62% replied that the absence of available robotic surgery harmed their surgical training. It is noteworthy that among residents from centers with available robotic surgery similar findings were observed—61% replied that the adoption of robotics harmed their training. Another interesting insight was that 72% of residents from centers with available robotic surgery answered that they would benefit from a rotation in open surgery at a center where robotics is not available. Moreover, 85% of residents from centers with no available robotics answered that they would benefit from a robotic rotation at neighboring wards.

Participation in post-graduate urology education was reported to be 82%. Even so, only 52% of them reported to have participated in more than 75% of these meetings.

As far as engaging in research, 57% stated that they had presented an abstract in the Israel Urologic Association annual meeting, and 21% stated that they had presented abstracts at international meetings.

Motivation for engaging in continued urologic training is high: 81% of the residents replied that they intend to pursue a clinical fellowship upon residency graduation. The most popular fields were uro-oncology and endo-urology fellowships (30% and 20%, respectively).

## DISCUSSION

The extent and role of general surgery training during urology residency are being constantly shortened. Once a subspecialty of general surgery, urology has become an independent discipline with unique fields requiring unique and different skills such as those required for endo-urology. In Israel, this rotation has been gradually shortened in the past years from 2 years to 9 months. A similar reduction from 2 years to 1 year of general surgery training has been carried out in the USA.^4^ Though most residents replied that the general surgery rotation has made a marginal contribution to their training, half replied that the duration should not be changed from the current 9 months. There is a current motion by the Israel Urology Association to abolish completely this 9-month mandatory general surgery rotation, and to allow instead an elective 3-month rotation. Another aspect to be addressed is the Step A examination in general surgery. Most residents replied that this examination is mostly irrelevant for their urology training. Even so, we believe that some core topics (e.g. fluid and electrolyte balance, trauma, intensive care) should be covered and tested, but this can be done in a separate section in the Step A urology examination.

The current surgical expertise required of practicing urologists is extensive and has steep learning curves. This makes surgical training of competent urologists very challenging. Two decades ago, urology surgeons would have mastered mostly open surgery and endoscopic surgery to some extent. Today the urologic surgical spectrum has expanded and shifted to include complex endoscopic surgery, laparoscopic surgery, robotic surgery, and more. Considering these changes, urologic surgical training during the residency requires hands-on training in a wide range of procedures. As shown in our survey, this is currently not evident in most residency programs in Israel. The residents’ perspective on the quality of the surgical training is disturbing. Most residents reported little surgical exposure, no hands-on training, and thus that the degree of confidence in performing complex procedures is generally low. The increase in degree of confidence in performing simple procedures (e.g. ureteroscopy, transurethral resections) does not continue, with advancing PGY, in the more complex procedures (e.g. radical prostatectomy, radical nephrectomy). Others have reported real-life low surgical competence of graduating residents in other fields.^5^

Since it is beyond the scope of this paper to discuss the entire urologic surgical arsenal, we chose to focus on minimally invasive surgery. As far as training in minimally invasive surgery goes, there is a shift from the traditional “see-one-do-one-teach-one” to a more staged and structured learning based on e-learning and modular training settings.^6^ A 2014 Cochrane meta-analysis showed that laparoscopic box-model training improves technical skills compared to no training among trainees with no prior laparoscopic skills.^7^ Tunc et al. reported significant improvement of laparoscopic suturing task during a 3-day dry and wet course.^8^ Klein et al. extrapolated that a step-wise laboratory course in vesico-urethral anastomosis (VUA) can bring an unexperienced trainee to performance skills comparable to those of a surgeon with the experience of 50 laparoscopic VUAs.^9^ Several courses and training sessions are available in laparoscopy. The European Training in Basic Laparoscopy (E-BLUS) by the European Association of Urology (EAU) offers several laparoscopy courses starting from basic skills training in a module (dry laboratory) to more complex courses using an animal model.^10^ A similar program has been developed and validated by the American Urologic Association (AUA)—the AUA BLUS (Basic Laparoscopy Urologic Skills).^11^ Further training can be gained using laparoscopic simulators, but a major flaw of these is the absence of tactile stimulation.^12^,^13^ Furriel et al. reported that most European urology residents regard their laparoscopic experience to be poor. They also acknowledged that residents’ access to laparoscopy laboratories and participation in laparoscopy courses were low despite a high motivation to gain skills in this field.^14^ In the USA, though a 5-fold increase was reported in the availability of virtual reality simulators in recent years, the frequency of use remained unchanged and the reported formal laparoscopic curricula decreased.^15^ In Japan, an interinstitutional nationwide assessment system covering many surgical subspecialties is employed. This system, called the Endoscopic Surgical Skill Qualification (ESSQ), was developed in 2004 and assesses applicants who are video-recorded while performing the entire procedure, and then assessed.^16^ By 2015, more than 8000 surgeons had completed the ESSQ—more than 2300 were urologists and 1331 of them qualified.^17^ Many of the training concepts regarding laparoscopic surgery can be applied to robot-assisted surgery. Virtual reality simulators have been efficacious in improving trainees’ skills.^18^ The EAU European Basic Robotic Urology Skills (E-BRUS) is the robotic equivalent of the E-BLUS.

To date, none of these training programs have been routinely and formally incorporated as obligatory in Israeli urology residency programs. It should be noted that some centers in Israel do employ local minimally invasive training initiatives, especially those of the “Clalit” health organization. Given the steep learning curves of laparoscopic surgery, formal laparoscopic training in the form of one or more of the above-mentioned courses can be incorporated as mandatory in the Israeli residency curriculum. Another quality end-point may be setting specific laparoscopic tasks to be mastered upon residency graduation. An example is being able to separate and mobilize the kidney in transperitoneal nephrectomy/partial nephrectomy. Similar milestones for specific procedures have been reported in other surgical fields.^19^

Labor regulations have been widely adopted in recent years. The “26-hour shift limit” or “60–80-hour working week” generated major changes in work routines in most medical disciplines. The obligatory day-off after on-call duties was introduced in Israel in 2000. Although arguments in favor of a limited working schedule have good reasoning—helping the exhausted young physician and lowering the odds of consequent treatment errors—they do not come without a cost. If a resident performs six monthly on-calls on average it can be estimated that 20%–25% of total residency time is lost compared with the former situation. A similar trend has been reported to result in a 20%–25% reduction in surgeons’ surgical volume in Europe.^20^ In contrast, recently the Accreditation Council for Graduate Medical Education (ACGME) extended the permissible work shift duration for first-year residents from 16 to 24 hours.^21^ This was mainly based on data that longer shifts did not translate to inferior patient outcomes or resident satisfaction.^22^ It can be postulated that the actual reduction in operating room time during the residency can partially explain the residents’ low perspective of their surgical expertise. In light of these changes the need for improving surgical training during the residency could not be overemphasized.

The introduction of robotic surgery has brought an obvious decline in the volume of open surgery similar to that seen with laparoscopy. As evident in the survey, more than half of the residents from centers with available robotics replied that this technology harmed their training. The first explanation for this observation is the above-mentioned volume decline in open surgery. Another explanation is the usual story of new technology adoption—whilst senior surgeons are still on their “learning curve,” surgical education of residents is left to one side and hence the residents’ apprehension that this technology has harmed their training. Practically, open radical retropubic prostatectomy is seldom carried out in centers with available robotic surgery. The same is true for radical cystectomy in some centers. Soon a day will come when surgeons facing the need for open conversion during a robotic procedure would have a problem due to lack of experience. Meanwhile, these procedures continue to be performed through the open approach in centers with no available robotic surgery. At the time of writing, 7 of the 17 approved urology programs in Israel offer robotic surgery. Naturally, training residents in these complex procedures is carried out with a single approach in each center. This unique situation can be well used to create surgical diversity during the urology training. A mandatory rotation in robotic surgery in a parallel urology ward could make a significant contribution to residents with no available robotics in their program. Similarly, open surgery rotations can be offered to residents with available robotics. Most residents replied they would like to engage in such rotations.

Most of the training in urology is done in the in-patient setting. However, a substantial part of the work of every practicing urologist is the out-patient clinic. Even so, less than half of non-junior residents (PGY3 and beyond) practice a routine clinic. The rates are considerably higher for PGY5 and PGY6, but still a substantial number of residents are not exposed to this activity. Setting a minimum period of practicing in a urology clinic in the residency curriculum should be considered.

Involvement and conducting research is a fundamental aspect in training top-end urologists. Most residents applying for a urology program in the USA value the opportunity to participate in research. Even so, only one-third are willing to “pay” an additional research year during their residency.^23^ It has been reported that publication output during urology residency predicted future academic achievements.^24^ An additional year dedicated to research during urologic residency in the USA was reported to translate to more than twice as many publications during the residency period (3 versus 7 total publications in 5-year and 6-year programs, respectively).^25^ About half of the residents reported having presented abstracts at an academic meeting in the survey. At the present time, the Israeli urology residency curriculum has a structured 6-month period of basic science during which the residents perform a research study and file a report. It is obvious that a 6-month period is too short for conducting serious research. Moreover, the fact that there is no publication requirement leads many residents to aim only for the necessary minimum. Thus, the added value of the current basic science period is often marginal and in many cases a waste of precious training time. An alternative to the current situation can be abandoning the obligatory 6-month period and proposing an optional research year only for residents who are interested.

## CONCLUSION

The urology “playground” has changed dramatically in past years. These fundamental changes have changed the skills required of a practicing urologist. The survey outlines the residents’ perspective on some of these changes. According to these perspectives we make suggestions for changes in the outdated current program. These include modifying the general surgery rotation and exam, optimizing surgical training, exchange rotations in robotic and open surgery, mandatory out-patient urology clinic exposure, and changes in the basic science period. We believe that implementing these changes would improve the current residency program and help train top-end urologists.

## Supplementary Material



## Abbreviations

ACGME: Accreditation Council for Graduate Medical Education
AUA: American Urological Association
BLUS-AUA: AUA Basic Laparoscopy Urologic Skills
EAU: European Association of Urology
E-BLUS: the European Training in Basic Laparoscopy
E-BRUS: European Basic Robotic Urology Skills
NAS: Next Accreditation System
PGY: post-graduate year
USA: United States of America
VUA: vesico-urethral anastomosis.
