# Supplementary material for: The Urology Residency Program in Israel—Results of a Residents Survey and Insights for the Future
Source: Rambam Maimonides Med J. 2017 Oct 16;8(4):e0039. doi: 10.5041/RMMJ.10317 (PMC5652930; doi:10.5041/RMMJ.10317)
Supplement: Supplementary file 1 [file rmmj-8-4-e0039-am.docx]

This appendix has been provided by the authors to give readers additional background reading

Supplement to The Urology Residency Program in Israel—Results of a Residents Survey and Insights for the Future

Lavi A, Tzemah S, Hussein A, Bishara I, Shcherbakov N, Zelichenko G, Mashiah A, Gross M, Cohen M. The Urology Residency Program in Israel—Results of a Residents Survey and Insights for the Future. *Rambam Maimonides Med J* 2017;8 (4):e0039. doi:10.5041/RMMJ.10317

# English Translation of the Web-Based Survey

1. **Where did you graduate medical school?**
2. Israel
3. Abroad
4. **What post graduate year are you?**

1 2 3 4 5 6 7

1. **How many residents are you in the ward?**

1 2 3 4 5 6 7 8 9

1. **How many night on-calls do you perform per month, on average?**

1 2 3 4 5 6 7 8 More than 8

1. **Have you taken the general surgery rotation?**
2. Yes
3. I’m performing it at the moment
4. No
5. **To what extent did the general surgery rotation contribute to your training?**

Very Little Very Much

1 2 3 4 5

1. **Do you believe that the general surgery rotation should be modified?**
2. It should be cancelled/shortened to an elective 3 months rotation
3. It should be shortened to a 6 months mandatory rotation
4. It should be shortened to a 3 months mandatory rotation
5. The rotation should be prolonged
6. **Do you feel that first step in general surgery contributed to your training?**
7. Yes
8. No
9. I don’t know
10. **How would you define the urological surgical exposure during the residency?**

Not Good Excellent

1 2 3 4 5

1. **To what extent would you define the surgical training in your program as ‘Hands on training’?**

Not At All Very Much

1 2 3 4 5

***Questions 11-13 are designated for post graduate year 3 and up residents only***

1. **How confident do you feel performing the following procedures independently?**
2. **TUR-BT**

Not Confident Very Confident

1 2 3 4 5

1. **TUR-P**

Not Confident Very Confident

1 2 3 4 5

1. **Simple Ureteroscopy (10mm> distal ureter stone)?**

Not Confident Very Confident

1 2 3 4 5

1. **Simple open Prostatectomy (Suprapubic/Retropubic)?**

Not Confident Very Confident

1 2 3 4 5

1. **Open Radical Nephrectomy (simple/radical)?**

Not Confident Very Confident

1 2 3 4 5

1. **Open Partial Nephrectomy?**

Not Confident Very Confident

1 2 3 4 5

1. **Laparoscopic Nephrectomy (simple/radical)?**

Not Confident Very Confident

1 2 3 4 5

1. **Laparoscopic Partial Nephrectomy?**

Not Confident Very Confident

1 2 3 4 5

1. **Open Radical Prostatectomy?**

Not Confident Very Confident

1 2 3 4 5

1. **Robotic assisted laparoscopic radical prostatectomy?**

Not Confident Very Confident

1 2 3 4 5

1. **Circumcision?**

Not Confident Very Confident

1 2 3 4 5

1. **Hydrocele?**

Not Confident Very Confident

1 2 3 4 5

1. **Performing and analyzing a urodynamic test?**

Not Confident Very Confident

1 2 3 4 5

1. **Do you practice routinely a urology out-patient clinic?^[[1]](#footnote-1)^**
2. Yes
3. No
4. Yes, but not routinely
5. **How many hours a week do you work in an out-patient clinic?**
6. 1–2
7. 2–5
8. 5–10

***Robotic Surgery***

1. **Is there a surgical robot in your institution?^[[2]](#footnote-2)^**
2. Yes
3. no

***For residents who answered ‘no’ to question 14***

**Do you feel that the absence of a robot is harming your training?**

1. Yes
2. No

**Would you like to be exposed to robotic surgery in a designated rotation?**

1. Yes
2. No

***For residents who answered ‘yes’ on question 14***

**In how many Robotic procedures do you take part monthly?**

1. 0
2. 0-5
3. 5-10
4. > 10

**Do you feel that the infusion of robotic surgery along with the decline in open surgery harmed your training?**

1. Yes
2. No

**Would you like to be exposed to open surgery in a designated rotation?**

1. Yes
2. No
3. **Do you take part in a post graduate education program?**
4. Yes, routinely (more than 75% of the meetings)
5. Yes, routinely (25%- 75% of the meetings)
6. Seldom (less than 25% of the meetings)
7. I don’t take part in these programs

***General Questions (continued)***

1. **Do you intend to persue a clinical fellowship at the end of the residency?**
2. Yes
3. No
4. I don’t know
5. **In what field do you want to perform your fellowship?**
6. Uro-oncology
7. Endo-urology
8. Pediatric Urology
9. Female Urology
10. Infertility
11. Reconstructive Urology
12. **How many conferences in Israel have you participated in during your residency?**
13. 1–2
14. 3–5
15. 5–10
16. >10
17. **Have you ever presented an abstract at the annual meeting of the Israeli Urologic Association?**
18. Yes
19. No
20. **Have you ever participated/presented in an international meeting (AUA/EAU)?**
21. Yes. I presented once
22. Yes. I presented several times
23. No
24. I participated without presenting

1. If answered positively, the participant was automatically directed to question #13 [↑](#footnote-ref-1)
2. Participants were automatically directed to the relevant set of questions, based on their answer. [↑](#footnote-ref-2)
